# Molecular epidemiology, clinical analysis, and genetic characterization of Zika virus infections in Thailand (2020–2023)

**DOI:** 10.1038/s41598-023-48508-4

**Published:** 2023-11-29

**Authors:** Sarawut Khongwichit, Watchaporn Chuchaona, Sompong Vongpunsawad, Yong Poovorawan

**Affiliations:** Center of Excellence in Clinical Virology, Faculty of Medicine, Chulalongkorn University, 1873 Rama 4 Road, Pathumwan, Bangkok, 10330 Thailand

**Keywords:** Genetics, Medical research, Epidemiology, Diseases, Infectious diseases, Microbiology, Virology, Viral epidemiology, Viral evolution, Evolution, Molecular evolution

## Abstract

To investigate the clinical and molecular characteristics and evolution of the Zika virus (ZIKV) in Thailand from March 2020 to March 2023. In all, 751 serum samples from hospitalized patients in Bangkok and the surrounding areas were screened for ZIKV using real-time RT-PCR. Demographic data and clinical variables were evaluated. Phylogenetic and molecular clock analysis determined the genetic relationships among the ZIKV strains, emergence timing, and their molecular characteristics. Among the 90 confirmed ZIKV cases, there were no significant differences in infection prevalence when comparing age groups and sexes. Rash was strongly associated with ZIKV infection. Our ZIKV Thai isolates were categorized into two distinct clades: one was related to strains from Myanmar, Vietnam, Oceania, and various countries in the Americas, and the other was closely related to previously circulating strains in Thailand, one of which shared a close relation to a neurovirulent ZIKV strain from Cambodia. Moreover, ZIKV Thai strains could be further classified into multiple sub-clades, each exhibiting specific mutations suggesting the genetic diversity among the circulating strains of ZIKV in Thailand. Understanding ZIKV epidemiology and genetic diversity is crucial for tracking the virus's evolution and adapting prevention and control strategies.

## Introduction

Zika virus (ZIKV) is a single-stranded RNA flavivirus primarily transmitted by the *Aedes* mosquitoes. It was first discovered in Uganda in 1947 and identified in Asia in 1966^1,2^. Prior to 2007, only sporadic ZIKV infection cases with self-limiting or mild symptoms were documented in Africa and Asia^3^. In 2007, the first ZIKV outbreak occurred in Yap Islands, Micronesia, affecting 73% residents^4^. Subsequent outbreaks occurred in French Polynesia in 2013–2014, during which the association between ZIKV infection and Guillain–Barré syndrome was noted^5,6^. ZIKV was first identified in Brazil in 2015^7^ and rapidly spread throughout the Americas^8^. Brazil experienced a dramatic rise in ZIKV-linked neonatal microcephaly cases, resulting in the declaration of a public health emergency of international concern by the WHO in early 2016 to establish a causal connection between ZIKV and congenital disabilities^9^. Since then, many countries have increased their focus on monitoring ZIKV infections.

Before 2016, multiple lines of evidence indicated that ZIKV circulated at low levels, and sporadic cases were reported in Southeast Asian countries including Thailand for decades^10^. From 2016 to 2017, the number of ZIKV infection cases in Thailand dramatically increased by over 1500 cases; however, it remains unclear whether this rise was because of higher infection rates or increased awareness^11^. According to the Bureau of Epidemiology, Ministry of Public Health, Thailand, the morbidity rate in 2016 was 1.69 per 100,000 population. From 2019 to 2022, the morbidity rates of ZIKV in Thailand were < 0.5 yearly; the rates were 0.41, 0.36, 0.10, and 0.29 per 100,000 population, respectively^12^. From 2016 to 2022, the Bureau reported 234 confirmed cases of ZIKV in pregnant women. Among them, 11 patients experienced miscarriages, of which four were related to ZIKV infection. Furthermore, clinical surveillance of 2217 neonates with microcephaly revealed 15 cases of congenital Zika syndrome.

While the ZIKV epidemic and its genetic characterization in the Americas are well documented, its presence and molecular epidemiology in Southeast Asia, particularly in Thailand, are areas of concern and ongoing investigation. Few studies have explored the molecular epidemiology of the Thai ZIKV strains. Hence, more research is required on the current genetic characterization and diversity of ZIKV strains in Thailand since the COVID-19 pandemic. This research aimed to comprehensively evaluate the ZIKV prevalence, clinical presentation, and genetic characteristics in Thailand from 2020 to 2023. Investigating the genetic diversity of the current ZIKV circulating in Thailand can help assess the risk of outbreaks and guide public health strategies and preparedness efforts.

## Results

### Demographic characteristics and clinical features

Out of the 751 samples (Table 1), 12.0% (90/751; 56.7% female and 43.3% male) tested positive for ZIKV infection based on Zika viral RNA presence. There was no significant sex-related difference in ZIKV prevalence (*p* = 0.507). The median age of patients with confirmed ZIKV was 37 (IQR: 29–46) years (range: 1–71 years). Most patients were in the 36–45 years age group (32.2%), followed by 26–35 years (22.2%) and 46–55 years (13.3%). Prevalence was lower among participants aged ≤ 15 years (10%) and 16–25 years (10%). Age was not significantly associated with increased ZIKV infection (*p* = 0.187). The median duration from illness onset to Zika RNA diagnosis was 3.5 days (IQR: 3–5 days).

**Table 1:** Demographic characteristics and clinical presentation of individuals according to ZIKV infection, Thailand (2020–2023) (N = 751).

Variable	All patients	ZIKV positive	ZIKV negative	OR (95% Cl)	*p*-value
Sex‒no./total no. (%)					
Female	401/751 (53.4)	51/90 (56.7)	350/661 (53.0)	Reference	0.507
Male	350/751 (46.6)	39/90 (43.3)	311/661 (47.0)	0.86 (0.55‒1.34)	
Age (years)					
Median (interquartile range)	35 (26‒47)	37 (29‒46)	34 (25–47)		
Range	1‒92	1‒71	1‒92		
Age distribution (no. [%])					
≤ 15 years	83 (11.1)	9 (10.0)	74 (11.2)	1.28 (0.49‒3.40)	0.187
16–25 years	104 (13.8)	9 (10.0)	95 (14.4)	Reference	
26–35 years	198 (26.4)	20 (22.2)	178 (26.9)	1.19 (0.52‒2.71)	
36–45 years	164 (21.8)	29 (32.2)	135 (20.4)	2.27 (1.03‒5.01)	
46–55 years	91 (12.1)	12 (13.3)	79 (12.0)	1.60 (0.64‒4.00)	
≥ 56 years	111 (14.8)	11 (12.2)	100 (15.1)	1.16 (0.46‒2.93)	
Days from illness onset to diagnosis					
Median (interquartile range)	4 (2–5)	3.5 (3–5)	3 (2–5)		
Symptoms—no./total no. (%)					
Fever	428/484 (88.4)	42/59 (71.2)	386/425 (90.8)	0.25 (0.13‒0.48)	< 0.001
Arthralgia	164/484 (33.9)	32/59 (54.2)	132/425 (31.1)	2.63 (1.52‒4.57)	< 0.001
Conjunctivitis	23/484 (4.8)	13/59 (22.0)	10/425 (2.4)	11.73 (4.87‒28.24)	< 0.001
Rash	133/484 (27.5)	49/59 (83.1)	84/425 (19.8)	19.89 (9.68‒40.90)	< 0.001
Myalgia	213/484 (44.0)	23/59 (39.0)	190/425 (44.7)	0.79 (0.45‒1.38)	0.407
Vomiting	71/484 (14.7)	3/59 (5.1)	68/425 (16.0)	0.28 (0.09‒0.93)	0.026
Diarrhea	33/484 (6.8)	2/59 (3.4)	31/425 (7.3)	0.45 (0.10‒1.91)	0.265
Headache	211/484 (43.6)	14/59 (23.7)	197/425 (46.4)	0.36 (0.19‒0.68)	0.001
Fatigue	132/484 (27.3)	14/59 (23.7)	118/425 (27.8)	0.81 (0.43‒1.53)	0.514
Sore throat	39/484 (8.1)	8/59 (13.6)	31/425 (7.3)	2.00 (0.87‒4.57)	0.098
Informed	484	59	425		
No informed	267	31	236		

The common clinical symptoms among ZIKV patients included rash (83.1%), fever (71.2%), arthralgia (54.2%), myalgia (39%), and conjunctivitis (22%). Skin rash was strongly associated with ZIKV infection (odds ratio [OR] 19.89, *p* < 0.001), as were arthralgia (OR 2.63, *p* < 0.001), and conjunctivitis (OR 11.73, *p* < 0.001). There was no evidence of ZIKV-associated neurological complications. Next, we examined the correlation between age groups and clinical characteristics and found that only arthralgia or joint pain (*p* = 0.022) showed a significant association with age groups (Table 2). In addition, the percentage of ZIKV-positive samples in each study year was analyzed and showed that there were 4.67% (12/257) tested positive for ZIKV infection in March 2020-December 2020, 7.54% (8/109) in 2021, 17.5% (47/269) in 2022. Interestingly, 19.8% (23/116) tested positive for ZIKV in the first three months of 2023.

**Table 2:** Clinical characteristics in different age groups of ZIKV-infected participants (N = 59).

Clinical characteristics	ZIKV-infected cases -no. (%)	Age distribution—no. (%)						*p*-value
		≤ 15 years (n = 8)	16‒25 years (n = 7)	26‒35 years (n = 12)	36‒45 years (n = 18)	46‒55 years (n = 9)	≥ 56 years (n = 5)	
Fever	42 (71.2)	7 (87.5)	6 (85.7)	10 (83.3)	9 (50.0)	7 (77.8)	3 (60.0)	0.262
Arthralgia	32 (54.2)	0 (0)	5 (71.4)	7 (58.3)	10 (55.6)	6 (66.7)	4 (80.0)	**0.022**
Conjunctivitis	13 (22.0)	0 (0)	3 (42.9)	4 (33.3)	3 (16.7)	2 (22.2)	1 (20.0)	0.370
Rash	49 (83.1)	7 (87.5)	7 (100.0)	10 (83.3)	15 (83.3)	6 (66.7)	4 (80.0)	0.713
Myalgia	23 (39.0)	0 (0)	3 (42.9)	4 (33.3)	7 (38.9)	5 (55.6)	4 (80.0)	0.061
Vomiting	3 (5.1)	1 (12.5)	0 (0)	1 (8.3)	1 (5.6)	0 (0)	0 (0)	0.884
Diarrhea	2 (3.4)	0 (0)	0 (0)	1 (8.3)	0 (0)	0 (0)	1 (20.0)	0.193
Headache	14 (23.7)	2 (25.0)	1 (14.3)	3 (25.0)	5 (27.8)	3 (33.3)	0 (0)	0.844
Fatigue	14 (23.7)	1 (12.5)	3 (42.9)	2 (16.7)	4 (22.2)	2 (22.2)	2 (40.0)	0.707
Sore throat	8 (13.6)	1 (12.5)	3 (42.9)	1 (8.3)	1 (5.6)	2 (22.2)	0 (0)	0.185

Numbers in bold represent statistically significant *p*-values.

### Genome sequence and phylogenetic analysis of ZIKV detected in Thailand during 2020–2023

We constructed a maximum likelihood phylogenetic tree and examined the nucleotide identity using complete coding sequences of ZIKV Thai strains of 2020–2023 from this study (n = 17) and additional sequences representing various strains sourced from the GenBank database. Our ZIKV Thai isolates belonged to the Asian lineage and could be classified into two clades: Southeast Asian (SEA) and Asian-American (AA).

**Figure 1:**
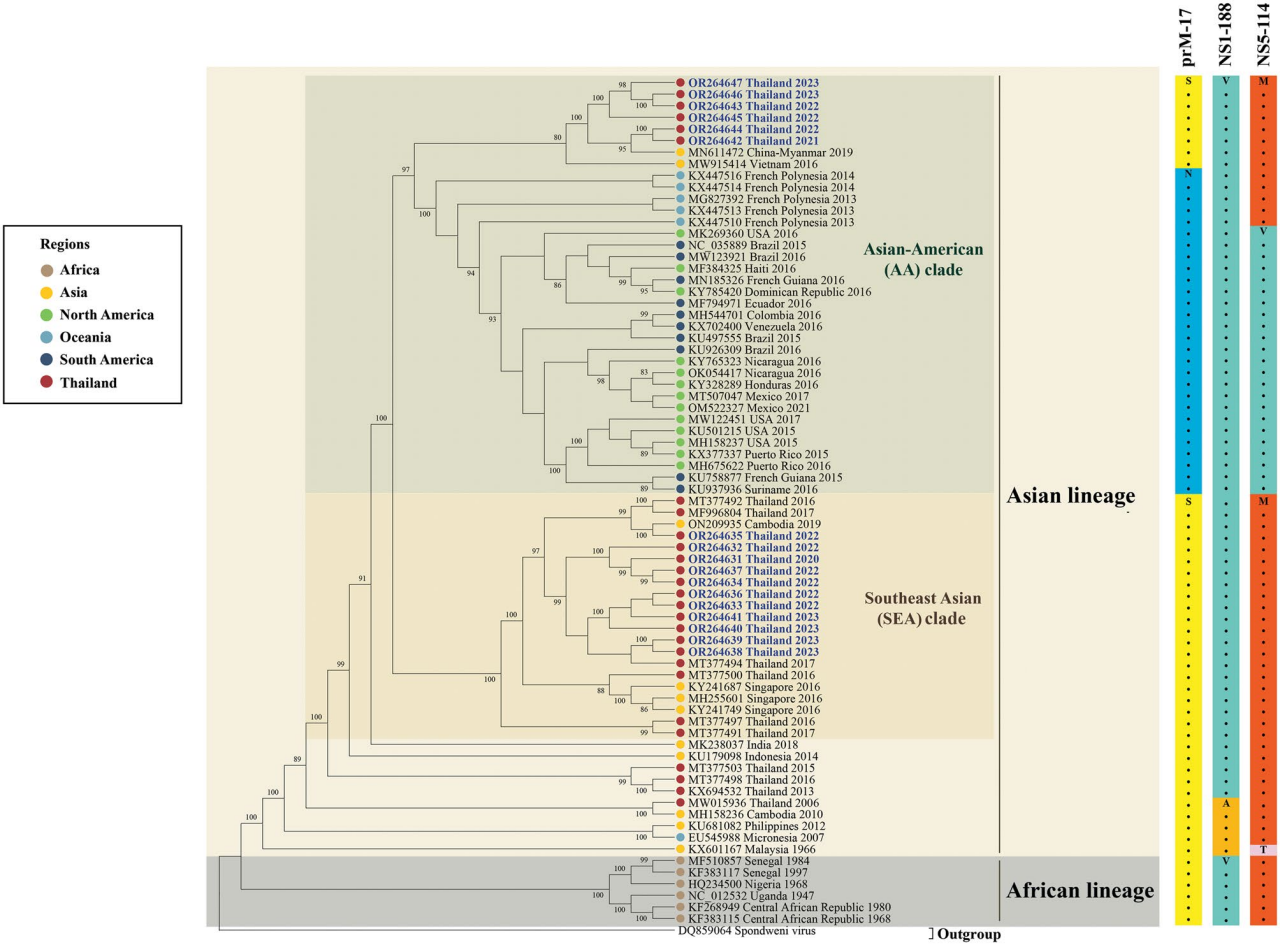
Phylogenetic tree analysis of the ZIKV complete coding sequence. The maximum-likelihood tree of ZIKV was constructed using the complete coding sequences of ZIKV Thai strains identified in this study and various strains from the GenBank database. The tree was generated using the GTR + I + G4 model with 1000 bootstrap replicates represented at the branch nodes. The two main clades of the ZIKV Asian lineage are highlighted in different colors. ZIKV strains isolated in this study are indicated in blue text (GenBank accession numbers OR264631-OR264647). Bold lines in different colors represent specific amino acid alterations in prM, NS1, and NS5.

**Figure 2:**
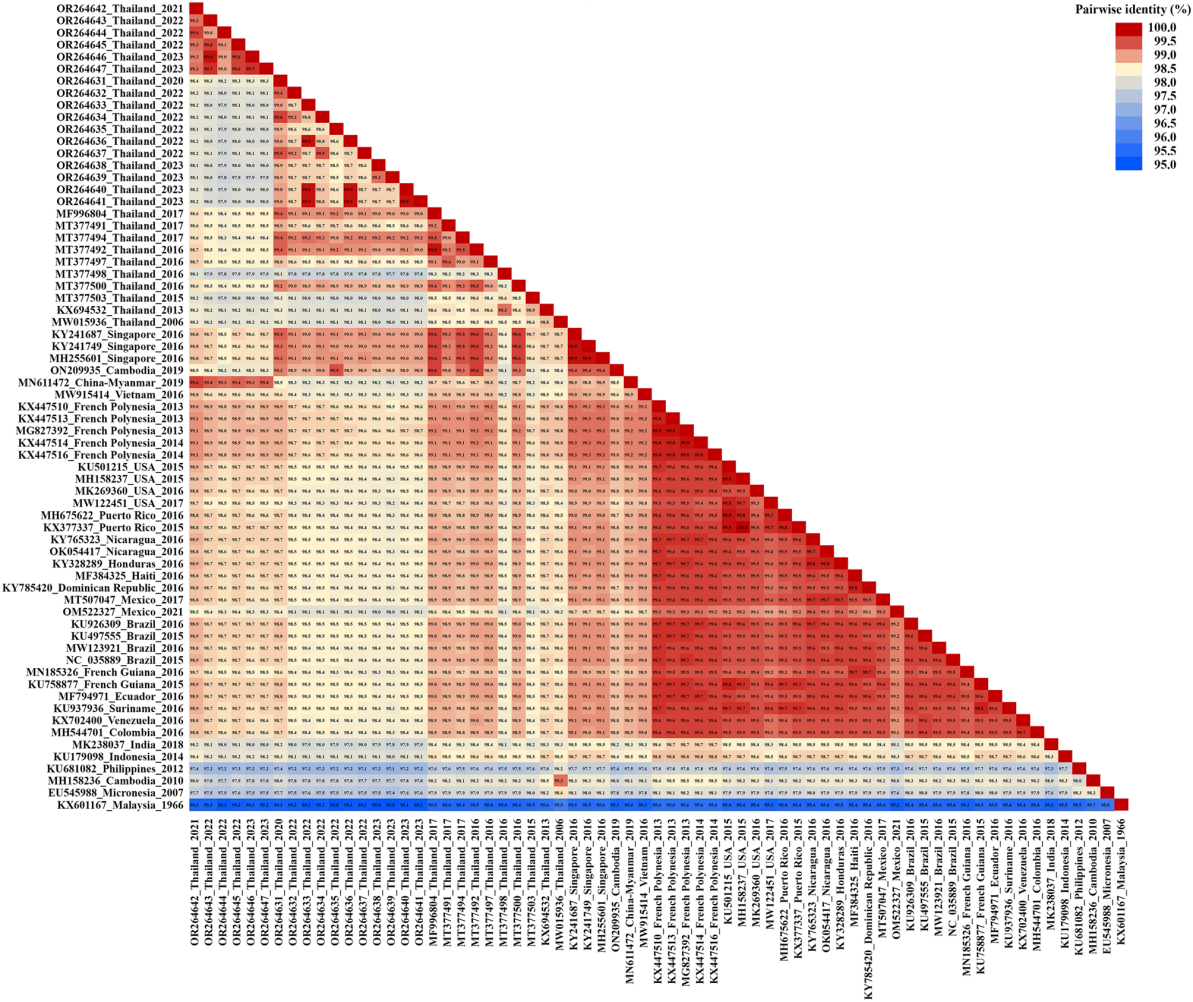
Nucleotide identity matrix of Zika virus. Values are indicated by color shading. A percent sequence identity matrix was generated from complete coding nucleotide sequences.

Out of the 17 ZIKV Thai isolates from 2020 to 2023 (Figs. 1 and 2), 11 were in the SEA clade, which includes strains from Thailand in 2016–2017 (98.5–99.4% sequence identity), Singapore in 2016 (99.0–99.4% sequence identity), and Cambodia in 2019 (98.8–99.5% sequence identity). Most of our SEA ZIKV strains were closely related to those from Thailand in 2017, while one of our SEA ZIKV (OR264635) detected in 2022 showed the highest nucleotide identity (99.5%) with the genome sequence of the virus detected in Cambodia.

In contrast, the remaining six isolates formed a cluster within the AA clade, related to viruses from China in 2019, Vietnam in 2016, French Polynesia in 2013–2014, and various countries across the Americas in 2015–2021. However, our AA Thai strains were more closely related to the viral genome obtained from a ZIKV-infected Chinese traveler who visited Myanmar in 2019 (99.3–99.6% sequence identity). A comparison of the nucleotide sequences between the complete coding genome of our AA ZIKV Thai strains and all previous ZIKV Thai strains of 2016–2017 showed 97.8–98.7% identity. Interestingly, ZIKV Thai strains of 2021–2023 in the AA clade showed that the level of nucleotide identity with ZIKV strains reported in French Polynesia from 2013 to 2014 (98.8–99.1%) was higher than the previous circulating strain of ZIKV in Thailand from 2006 to 2017 (97.8–98.7%). Meanwhile, our AA ZIKV Thai strains shared 98.3–98.9% nucleotide sequence similarity with ZIKV collected from North America and 98.5–98.9% nucleotide identity with ZIKV from different countries in South America.

Previous research divided the Asian lineage ZIKV into four genotypes based on amino acid substitutions at three specific positions: residue 17 in prM, residue 188 in NS1, and residue 114 in NS5. These genotypes are SAM, SVM, NVM, and NVV^13^. Our findings showed that Thai isolates and strains from other Southeast Asian countries have consistently belonged to the SVM genotype since 2013. In contrast, previous strains from Southeast Asian countries and Micronesia were primarily classified as the SAM genotype. The virus outbreaks across Oceania, such as French Polynesia, were NVM, while those from the Americas were NVV.

Our results suggested that ZIKV circulating in Thailand from 2020 to 2023 was caused by previous circulating strains within the country and neighboring countries such as Myanmar and Cambodia rather than being imported from French Polynesia and the Americas.

### Evolution of the ZIKV Asian lineage

**Figure 3:**
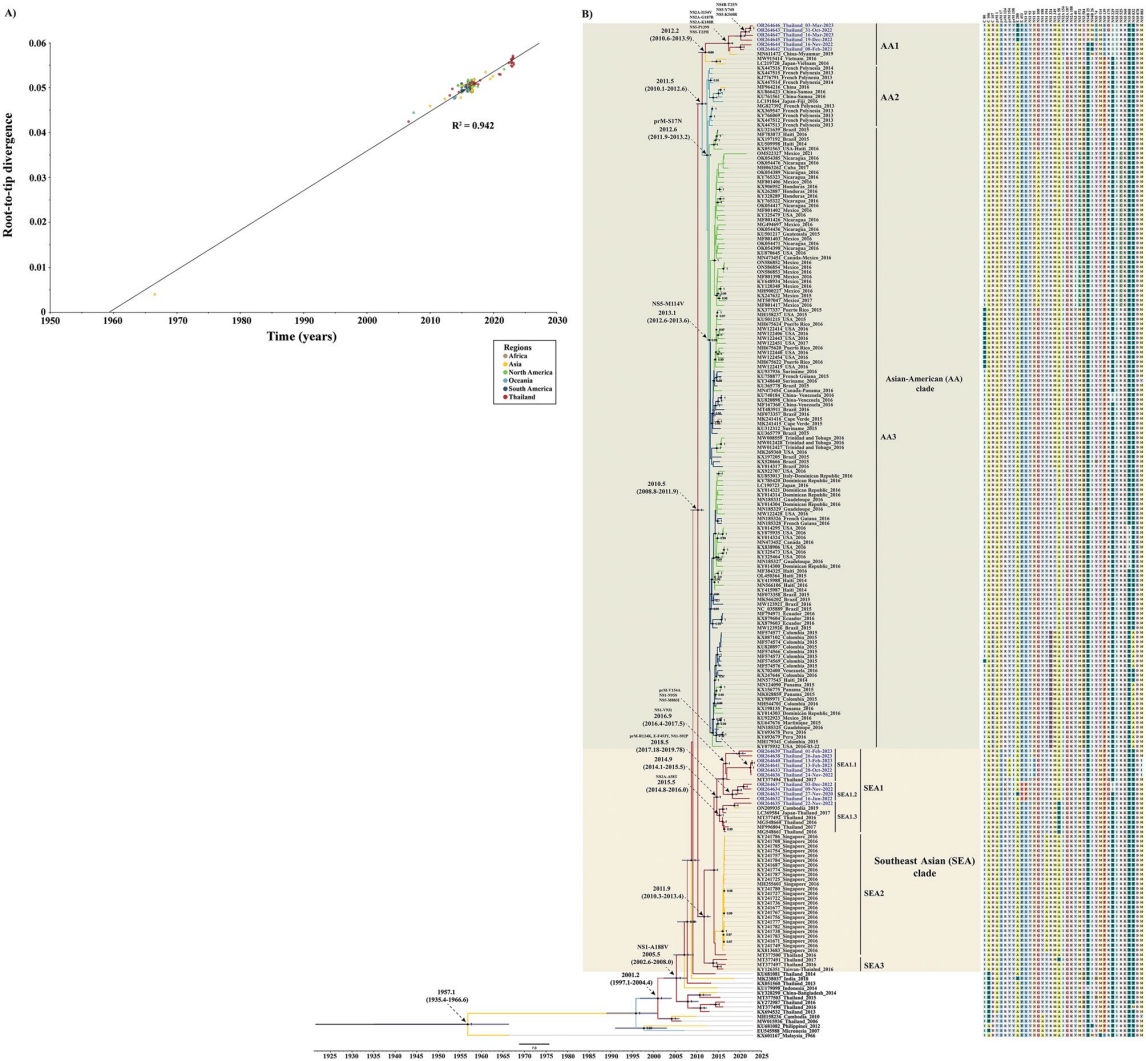
Molecular clock analysis of Asian ZIKV ancestry. (**A**) Temporal signal analysis of root-to-tip divergence regression versus date (R^2^ = 0.942). Maximum clade credibility (MCC) tree for the Asian ZIKV lineage. The most recent common ancestor (tMRCA) values with 95% HPD range and amino acid substitutions are represented by arrows. The black nodes are only displayed when the posterior probability (PP) > 0.95, while the blue node bars represent the 95% HPD values of the node height. The Thailand sequences discovered in this study (GenBank accession numbers OR264631-OR264647) are colored blue. Sequences are named using the format of accession number_country_collection year. The color of the branches in the tree corresponds to geographic regions, as indicated in the middle left of the MCC tree. The amino acid mutations specific to each clade or sub-clades of the Asian lineage are indicated next to the sequence tips of the MCC tree in panel (**B**).

To understand the evolution of the recent ZIKV Thai strains in this study and their relationships with previous Thai strains and other Asian-lineage viruses from various regions, we created a dataset consisting of 214 full-length coding sequences. We then constructed an initial Maximum Likelihood (ML) tree for root-to-tip analysis. This analysis revealed a strong positive temporal signal with an R^2^ value of 0.942 (Fig. 3A). Subsequently, we generated a time-calibrated maximum clade credibility (MCC) tree using Bayesian skyline plot inference (Fig. 3B).

The time-calibrated MCC tree analysis estimated the most recent common ancestor (tMRCA) for the Asian lineage of ZIKV to be in February 1957, with a 95% highest posterior density (HPD) between May 1935 and July 1966. The substitution rate was calculated at 7.95 × 10^−4^ substitutions per site per year (s/s/y). The tMRCA for the appearance of ZIKV in Thailand was estimated to be in March 2001, with a 95% HPD between February 1997 and May 2004. Notably, the NS1-A188V substitution, initially observed in ZIKV detected in Thailand in 2013, was found to have emerged around July 2005, with a 95% HPD spanning from August 2002 to January 2008. We also observed that ZIKV from Southeast Asia served as ancestors to the epidemic strains of French Polynesia and the other Asian ZIKV strains from the Americas.

Using timescale MCC tree analysis, we found that the Asian lineage virus diverged into the two main clades—SEA and AA—in June 2010, with a 95% HPD between October 2008 and November 2011, with a posterior probability (PP) of 1. The estimated tMRCA for the monophyletic SEA clade, which includes the ZIKV genome sequence from Thailand, Cambodia, and Singapore, was from December 2011, with an interval of April 2010 to June 2013. Three SEA subclades were observed, which comprise SEA1, SEA2, and SEA3. All of the ZIKV SEA Thai strains identified in this study belonged to SEA1, which emerged in November 2014 with a 95% HPD between February 2014 and July 2015. Nonsynonymous mutation divided our SEA1 Thai strains into sub-clades SEA1.1 (OR264633, OR264636, OR264638-264641), SEA1.2 (OR264631, OR264632, OR264634, OR264637), and SEA1.3 (OR264635), with tMRCAs around 2016.94, 2018.5, and 2015.50, respectively. Within sub-clade SEA1.1, all viruses exhibited a NS1-V93I substitution, four of which (OR264633, OR264636, OR264640, OR264641) shared three unique substitutions, namely prM-V154A, NS1-N95S, and NS5-M883I. In sub-clade SEA1.2, four Thai strains (OR264631, OR264632, OR264634, OR264637) contained three unique amino acid substitutions: prM-R124K, E-F453Y, and NS1-S92P. Remarkably, one ZIKV Thai strain from 2022 (OR264635) in sub-clade SEA1.3 shared the NS2A-A58T substitution with ZIKV Thai strains from 2016 to 2017 detected from cases of neurologic complication and ZIKV in Cambodia in 2019.

The tMRCA for the AA clade was estimated to be in June 2011, with a 95% HPD between February 2010 and July 2012. The AA clade was classified into three main sub-clades. Sub-clade AA1 contained six ZIKV Thai strains (OR264642, OR264643, OR264644, OR264645, OR264646, and OR264647), ZIKV isolated from the Chinese traveler returning from Myanmar in 2019, and ZIKV Vietnamese strains. The tMRCA for sub-clade AA1 was estimated to be in March 2012 (95% HPD: August 2010–November 2013). All ZIKV Thai strains in sub-clades AA1.1 shared five substitutions: NS2A-I154V, NS2A-G187R, NS2A-K188R, NS5-P139S, and NS5-T229I with the ZIKA strain originating in Myanmar in 2019. Four ZIKV Thai strains in sub-clades AA1.1 exhibited unique NS4B-T25N, NS5-Y74S, and NS5-K560R substitutions. Sub-clade AA2 contained ZIKV from Oceania (French Polynesia, Fiji, Samoa) and China. These strains had the amino acid substitution prM-S17N, initially observed in ZIKV from French Polynesia in 2013. The tMRCA for ZIKV with prM-S17N was dated approximately August 2012 (95% HPD between November 2011 and March 2013). Sub-clade AA3 contained ZIKV from the Americas, all possessing the amino acid substitution NS5-M114V. The tMRCA of strains from the Americas was estimated to be around April 2013, with a 95% HPD between July 2012 and July 2013.

## Discussion

This study investigated the demographic characteristics and clinical features related to ZIKV infection in Thailand since the COVID-19 pandemic. Our research also provides valuable insights into the epidemiology, genetic characteristics, and evolution of ZIKV in Thailand from March 2020 to March 2023. Among 751 hospitalized participants in Bangkok and the surrounding region who initially tested negative for both DENV RNA and CHIKV RNA and had negative CHIKV IgM results, 12% (90/751) were subsequently confirmed to have ZIKV infection based on Zika viral RNA detection. During the same period, the Bureau of Epidemiology, Ministry of Public Health, Thailand, reported 534 cases of ZIKV infection from approximately 20 of 77 provinces in Thailand^12^. Our study found ZIKV infection in 16.8% of cases documented by the Bureau between March 2020 and March 2023. In the first 3 months of 2023, we found 23 ZIKV-positive cases out of 90, accounting for around 53% of all confirmed Zika infections in Thailand reported by the Bureau. These findings suggest that Zika cases in the country are underreported and underdiagnosed. The restricted resources for ZIKV diagnostic tests may hinder active epidemiological surveillance and the inclusion of Zika virus disease into regular acute febrile illness tests. ZIKV infection causes non-specific symptoms like in chikungunya and dengue and hence, a significant proportion of asymptomatic ZIKV infections contributes to underdiagnosis and underreporting^3^.

Here, the clinical presentation of ZIKV infection was consistent with well-documented manifestations including fever, arthralgia, rash, conjunctivitis, and myalgia^14^. However, we found that rash was strongly associated with ZIKV infection, highlighting its significance as a critical clinical indicator. Similarly, the rash was observed in approximately 90% of ZIKV-infected individuals during outbreaks on Yap Island (2007), in French Polynesia (2013–2014), and Brazil (2015)^15,16^. Notably, our study found no evidence of ZIKV-related neurological complications. We found that the demographic distribution of ZIKV cases showed no significant association with age, although the highest prevalence was among individuals aged 36–45 years. However, our study revealed that this virus affects individuals across a wide age range, from children to older adults, emphasizing the importance of a surveillance system covering all age cohorts, as ZIKV can affect individuals of any age.

Our genetic analysis reveals that the ZIKV Asian lineage in Thailand from 2020 to 2023 is divided into two main clades: Asian-American (AA) and Southeast Asian (SEA). Our ZIKV Thai strains in the AA clade share genetic similarities with strains from Myanmar in 2019, Vietnam in 2016, French Polynesia in 2013–2014, and various American countries from 2015 to 2021. While most of our SEA Thai strains were closely related to previous circulating Thai strains from 2016 to 2017, one of our ZIKV SEA Thai strains (OR264635) showed the closest genetic affinity with a neurovirulent ZIKV strain isolated from Cambodia in 2019^17^, and a ZIKV Thai strain was linked to cases of congenital microcephaly^18^. Zhang et al.^17^ reported that the ZIKV strain from Cambodia in 2019 exhibited significantly higher neurovirulence in newborn mice, indicated by a 74-fold decrease in the 50% lethal dose (LD50), and led to markedly higher viral loads in neonatal mouse brains compared to the Cambodian strain from 2010. Similar to our study, a previous phylogenetic analysis of 79 partial NS5 sequences of the ZIKV Asian lineage collected from mosquitoes in Thailand in 2016 classified them into two distinct clades. One clade was related to ZIKV strains detected in the Americas, while the other was closely associated with ZIKV strains identified in Thailand from 2013 to 2017^19^.

The time-scale phylogeny of the Asian lineage revealed its likely introduction to Southeast Asia between May 1935 and July 1966. This estimate is supported by the evidence of neutralizing antibodies to ZIKV in Southeast Asian countries during the 1950s^10,20^. In Thailand, the possible presence of Zika was initially described in 1954 based on a serological survey^10^. However, Zika-neutralizing antibody-positive samples may have resulted from cross-neutralization owing to preexisting anti-DENV antibodies. Our estimated tMRCA suggests that ZIKV was first introduced to Thailand in the early 2000s.

The amino acid variations at positions 17 in prM, 188 in NS1, and 114 in NS5 have been essential in categorizing Asian lineage ZIKV isolates into four main genotypes—SAM, SVM, NVM, and NVV^13^. Our study identified ZIKV Thai strains of 2020–2023 as belonging to the SVM genotype, consistent with earlier findings in Thailand and Southeast Asian countries. Liu et al.^21^ proposed that changing alanine to valine at position 188 in the ZIKV NS1 protein (NS1-A188V) potentially enhances ZIKV transmission in mosquito vectors. Moreover, this variant has been found to enhance ZIKV replication by inhibiting interferon-β induction^22^. We found that the NS1-A188V mutation was first observed in a Southeast Asian country, with our tMRCA estimates indicating its emergence between August 2002 and January 2008, suggesting that the NS1-A188V mutation likely circulated within Southeast Asia for approximately 5–11 years before spreading to French Polynesia and the Americas.

None of the ZIKV strains circulating in the Asian region, including our recent Thai strains of 2020–2023 and French Polynesia have the substitution of methionine to valine at residue 114 in NS5 protein (M114V) or position 2634 from the start codon of the genome. Whereas all viruses circulating in the Americas exhibit NS5-114 V. Peng et al.^23^ found that the NS5-M114V mutation has negligible impact on enhancing the ability of ZIKV to replicate and spread. NS5-114 V is the signature of all American isolates but may not be involved in the outbreak. Our tMRCA estimates indicate that the ZIKV with NS5-M114V entered the Americas in April 2013. Consistent with these findings, a previous study has indicated that ZIKV probably entered Brazil in 2013, over a year before the identification of the initial outbreak in the Americas^24^.

Previous studies have shown that the prM-S17N alteration significantly enhances ZIKV replication in neural progenitor cells, induces severe microcephaly in mouse fetuses, and increases mortality in newborn mice^25^. The tMRCA for the prM-S17N mutant virus was estimated in late 2012, before the large ZIKV outbreak in French Polynesia from 2013 to 2014. The prM-S17N alteration in ZIKV was initially observed in the French Polynesian strain of 2013. Subsequently, this alteration has been consistently found in all ZIKV isolates from the Americas. However, it has not been detected in Asian strains, including the present Thai strains from 2020 to 2023, indicating that the prM-S17N mutant virus likely originated in French Polynesia before spreading to the Americas, but not Thailand.

Despite the absence of the S17N neurovirulent substitution in all ZIKV Thai strains, research by Wongsurawat et al.^18^ revealed that the endemic ZIKV strain in Thailand can lead to congenital ZIKV infection and microcephaly. A recent study described a case of a pregnant French woman who experienced an infection when traveling to Thailand at the end of 2021 during the first trimester of pregnancy, leading to severe brain abnormalities in the fetus^26^. Genetic analysis confirmed that this ZIKV strain lacked the neurovirulent S17N substitution. The occurrence of microcephaly in this report highlights the ongoing health risk posed by ZIKV in Thailand, even with a relatively low incidence in 2021. Notably, both reports underscore the potential for ZIKV in Thailand to induce microcephaly. While specific viral genetic factors can influence ZIKV-induced microcephaly, the stage of the host's pregnancy at the time of infection is critical in determining the severity of outcomes^27^.

In this study, we also noticed the number of nonsynonymous mutations in the present genome of ZIKV Thai strains. The viruses were classified into at least four sub-clades. Our results show the variation of ZIKV circulating in Thailand. However, the contribution of various mutations found in the Thai strains of 2020–2023 remains unknown. Therefore, further research is required to determine the significance of these mutations.

## Materials and methods

### Ethical approval statement

The research protocol for this study was approved by the Ethical Committee of the Faculty of Medicine, Chulalongkorn University, Thailand, under the institutional review board (approval number IRB710/64). All patient information and identifiers were anonymized to safeguard patient confidentiality. The institutional review board of the Ethics Committee for Human Research granted a waiver for written informed consent because all clinical specimens were anonymized. All experiments conducted in this study adhered to the relevant guidelines and regulations.

### Sample collection

Serum samples were obtained from 751 individuals with fever (temperature > 38.5 °C) or were suspected of mosquito-borne infection following the Pan American Health Organization guidelines^28^. The focus of this study was ZIKV mono-infection; therefore, patients with laboratory-confirmed chikungunya (positive nucleic acid test and/or IgM) or laboratory-confirmed dengue infection (positive nucleic acid test) were excluded. Samples were collected from five different provinces in Thailand from March 2020 to March 2023, including Bangkok, Samut Prakan, Samut Sakhon, Ratchaburi, and Chon Buri (Supplementary Fig. 1). In this study, a confirmed case of ZIKV infection was defined as a suspected mosquito-borne infection (fever > 38.5 °C with or without rash, myalgia, arthralgia, and conjunctivitis) plus laboratory confirmation by real-time reverse transcription PCR (RT-PCR) to detect ZIKV RNA.

### Detection of ZIKV and sequencing

RNA was extracted from serum samples by using the magLEAD® kit, followed by real-time RT-PCR using ZIKV-specific primers (ZIKV_1086: 5′-CCGCTGCCCAACACAAG-3′ and ZIKV_1162c: 5′-CCACTAACGTTCTTTTGCAGACAT-3′) and a fluorescent reporter dye probe (ZIKV_1107-FAM: 5′-AGCCTACCTTGACAAGCAGTCAGACACTCAA-3′), as described previously^29^. The sensitivity limit for the assay was 25 copies for the envelope gene target.

We then selected ZIKV-positive samples with a PCR cycle threshold (Ct) value less than 30 for genome sequencing. The complete coding sequence of ZIKV was amplified using specific sets of primers and PCR conditions as previously described^30^. The obtained amplicons were purified by gel-extraction purification (GeneAll Biotechnology, South Korea) before Sanger sequencing (First Base Laboratories, Selangor Darul Ehsan, Malaysia). Nucleotide sequence output was analyzed using the Basic Local Alignment Search Tool (BLAST) (http://blast.ncbi.nlm.nih.gov/Blast.cgi) and was edited by CHROMAS LITE v. 2.6.6 software. Sequence assembly was performed using BioEdit version 7.2.0. The nucleotide sequences were deposited in the GenBank database under the accession numbers OR264631-OR264647.

### Phylogenetic and Bayesian evolutionary analysis

The phylogenetic tree of the complete coding genome was constructed via the maximum-likelihood (ML) method with 1000 bootstrap replicates under the General Time Reversible (GTR) model with gamma distribution and proportion of invariable site parameters in MEGA 11 software. In this study, the complete coding genome sequences (total length 10,272 nucleotides) included the regions encoding three structural proteins (capsid, pre-membrane, and envelope) and seven non-structural (NS) proteins (NS1, NS2A, NS2B, NS3, NS4A, NS4B, NS5).

For further insights into the viral evolution, we created a dataset of ZIKV Asian lineage sequences from past outbreaks to the most recent epidemic. We assessed the temporal signal by analyzing root-to-tip divergence in our ZIKV sequences using TempEst v1.5.3 to ensure data suitability for time-scaled phylogenetic analysis.

The time-scaled phylogeny of the ZIKV complete coding genome was reconstructed using the Bayesian Markov Chain Monte Carlo (MCMC) approach with Bayesian Evolutionary Analysis Sampling Trees (BEAST) v1.10.4. Prior to this, we employed the SRD06 nucleotide substitution model, an uncorrelated lognormal relaxed clock, and the coalescent Bayesian skyline tree. Bayesian MCMC analyses were run in triplicate for 200 million generations, sampling every 10,000 generations. Convergence and effective sampling size (ESS) values (> 200) of parameters were assessed in Tracer v1.7.2.

To generate the time-scaled maximum clade credibility (MCC) tree, we combined the results of the three runs using LogCombiner v1.10.4, discarding the first 10% as burn-in. The resulting MCC tree was visualized with FigTree v.1.4.4.

### Data analysis

Qualitative data are presented as n (%) and the median with interquartile range for patient age and day of disease onset detection. The chi-square test and logistic regression analysis were used to assess demographic characteristics and the occurrence of symptoms among different groups. The statistical significance was assessed using SPSS software version 22 (IBM Corporation, Armonk, NY, USA). *p* < 0.05 was considered to indicate statistically significant differences.

### Supplementary Information

Supplementary Figure 1.

## Data Availability

All data produced during this study are contained within this article and its Supplementary Information files. The sequences produced in this work can be found in the GenBank database with the accession numbers OR264631-OR264647.

## References

[1] Dick GW, Kitchen SF, Haddow AJ (1952). Zika virus. I. Isolations and serological specificity. Trans. R. Soc. Trop. Med. Hyg..

[2] Marchette NJ, Garcia R, Rudnick A (1969). Isolation of Zika virus from *Aedes*
*aegypti* mosquitoes in Malaysia. Am. J. Trop. Med. Hyg..

[3] Plourde AR, Bloch EM (2016). A literature review of Zika virus. Emerg. Infect. Dis..

[4] Duffy MR (2009). Zika virus outbreak on Yap Island, Federated States of Micronesia. N. Engl. J. Med..

[5] Musso D, Nilles EJ, Cao-Lormeau VM (2014). Rapid spread of emerging Zika virus in the Pacific area. Clin. Microbiol. Infect..

[6] Oehler E (2014). Zika virus infection complicated by Guillain-Barre syndrome–case report, French Polynesia, December 2013. Eurosurveillance.

[7] Campos GS, Bandeira AC, Sardi SI (2015). Zika virus outbreak, Bahia, Brazil. Emerg. Infect. Dis..

[8] Hennessey M, Fischer M, Staples JE (2016). Zika virus spreads to new areas—region of the Americas, May 2015–January 2016. MMWR Morb. Mortal. Wkly. Rep..

[9] Gulland A (2016). Zika virus is a global public health emergency, declares WHO. Bmj.

[10] Pond WL (1963). Arthropod-borne virus antibodies in sera from residents of South-East Asia. Trans. R. Soc. Trop. Med. Hyg..

[11] Khongwichit S, Wikan N, Auewarakul P, Smith DR (2018). Zika virus in Thailand. Microbes Infect..

[12] Bureau of Epidemiology DDC, M., Thailand. Annual incidence report of Zika virus in Thailand. Accessed 20 April 2022. https://ddc.moph.go.th/dvb/ or http://doe.moph.go.th/surdata/disease.php?dcontent=old&ds=87.

[13] Liu Z-Y, Shi W-F, Qin C-F (2019). The evolution of Zika virus from Asia to the Americas. Nat. Rev. Microbiol..

[14] Lobkowicz L (2020). The frequency and clinical presentation of Zika virus coinfections: a systematic review. BMJ Glob. Health.

[15] Mo Y, Alferez Salada BM, Tambyah PA (2016). Zika virus—A review for clinicians. Br. Med. Bull..

[16] Cerbino-Neto J (2016). Clinical manifestations of Zika virus infection, Rio de Janeiro, Brazil, 2015. Emerg. Infect. Dis..

[17] Zhang Y-F (2023). Characterization and phylogenetic analysis of a neurovirulent Zika virus isolated from Cambodia in 2019. J. Med. Virol..

[18] Wongsurawat T (2018). Case of microcephaly after congenital infection with Asian Lineage Zika virus, Thailand. Emerg. Infect. Dis..

[19] Phumee A (2019). Molecular epidemiology and genetic diversity of Zika virus from field-caught mosquitoes in various regions of Thailand. Pathogens.

[20] Hammon WM, Schrack WD, Sather GE (1958). Serological survey for a arthropod-borne virus infections in the Philippines. Am. J. Trop. Med. Hyg..

[21] Liu Y (2017). Evolutionary enhancement of Zika virus infectivity in *Aedes*
*aegypti* mosquitoes. Nature.

[22] Xia H (2018). An evolutionary NS1 mutation enhances Zika virus evasion of host interferon induction. Nat. Commun..

[23] Peng NYG (2022). The distinguishing NS5-M114V mutation in American Zika virus isolates has negligible impacts on virus replication and transmission potential. PLoS Negl. Trop. Dis..

[24] Faria NR (2016). Zika virus in the Americas: Early epidemiological and genetic findings. Science.

[25] Yuan L (2017). A single mutation in the prM protein of Zika virus contributes to fetal microcephaly. Science.

[26] Marquine S (2023). Sequence data from a travel-associated case of microcephaly highlight a persisting risk due to Zika virus circulation in Thailand. J. Infect. Dis..

[27] Zorrilla CD, García García I, García Fragoso L, De La Vega A (2017). Zika virus infection in pregnancy: Maternal, fetal, and neonatal considerations. J. Infect. Dis..

[28] Pan American Health, O. & Pan American Health, O. (PAHO, 2022).

[29] Lanciotti RS (2008). Genetic and serologic properties of Zika virus associated with an epidemic, Yap State, Micronesia, 2007. Emerg. Infect. Dis. J..

[30] Leguia M (2017). Full-genome amplification and sequencing of Zika viruses using a targeted amplification approach. J. Virol. Methods.

