# Supplementary figures and images for: Molecular epidemiology, clinical analysis, and genetic characterization of Zika virus infections in Thailand (2020–2023)
Source: Sci Rep. 2023 Nov 29;13:21030. doi: 10.1038/s41598-023-48508-4 (PMC10687007; doi:10.1038/s41598-023-48508-4)

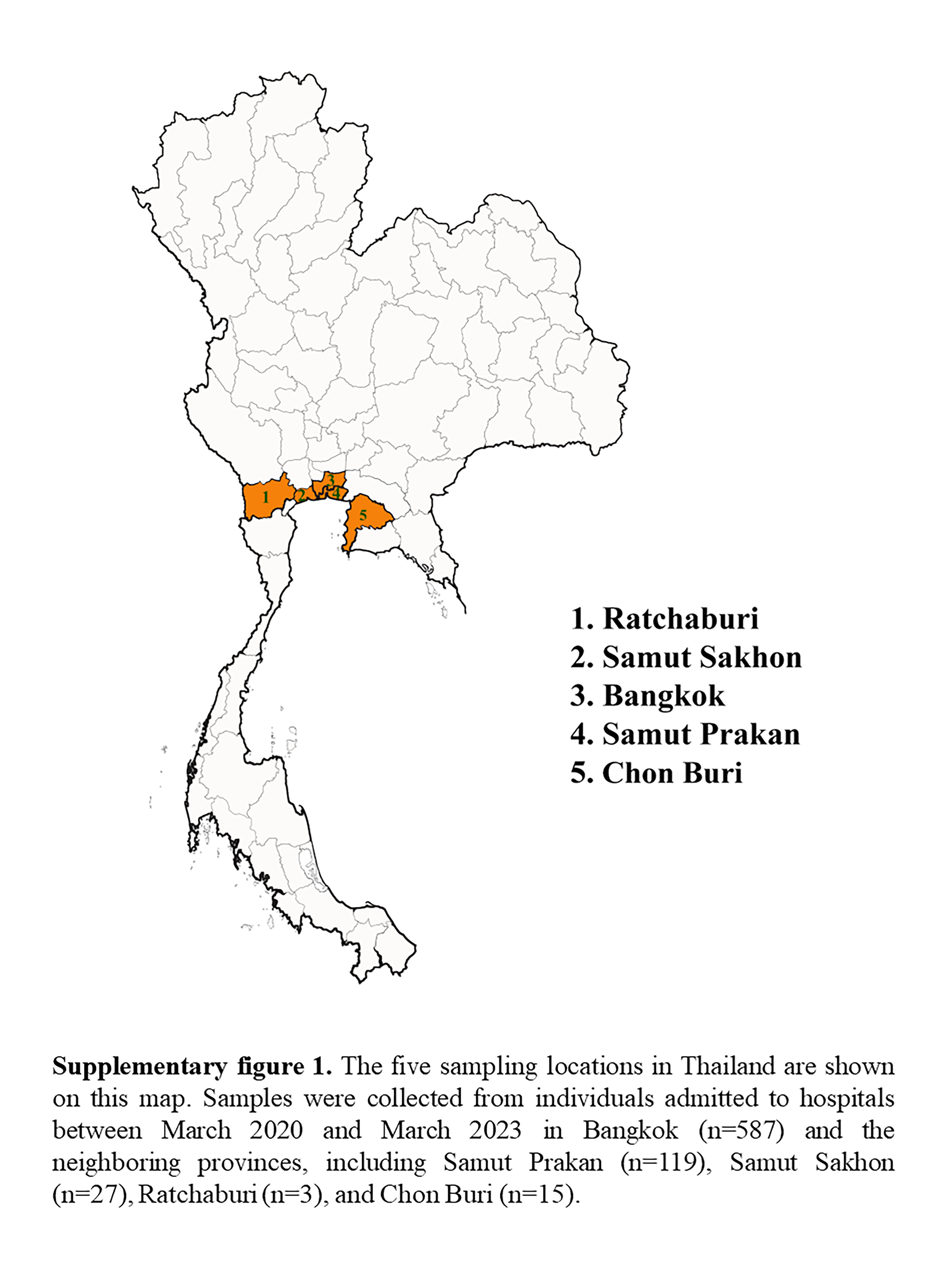

Supplement: Supplementary file 1 — Supplementary Figure 1. [file 41598_2023_48508_MOESM1_ESM.tif]
